# Glycyrrhizic acid induced acquired apparent mineralocorticoid excess syndrome with a hyperadrenergic state: a case report

**DOI:** 10.1186/s13256-024-04674-1

**Published:** 2024-08-07

**Authors:** John Szendrey, Anthony Poindexter, Gregory Braden

**Affiliations:** 1 Department of Internal Medicine, UMass Chan Medical School, Baystate Medical Center, 759 Chestnut St., Springfield, MA, 01199 USA; 2 Division of Nephrology, UMass Chan Medical School, Baystate Medical Center, 759 Chestnut St., Springfield, MA, 01199 USA

**Keywords:** Apparent mineralocorticoid excess, Glycyrrhizic acid, Nutritional supplement, Hypertension, Case report

## Abstract

**Background:**

Syndrome of apparent mineralocorticoid excess (AME) is characterized by excessive MR stimulation despite low levels of aldosterone. 11Beta-hydroxysteroid dehydrogenase-2 (11βDSH-2) inactivates cortisol to cortisone, preventing cortisol-induced MR activation. Genetic defects in 11βDSH-2 cause AME through accumulation of cortisol in the distal nephron, leading to MR activation induced hypertension, hypokalemia and metabolic alkalosis. Acquired AME can occur due to the ingestion of glycyrrhizic acid, found in licorice root, which inhibits 11βDSH-2 and has additional effects on cortisol homeostasis through inhibition of 11βDSH-1.

**Case report:**

We present a case of acquired AME with a hyperadrenergic symptoms induced by ingestion of Advanced Liver Support, a nutritional supplement produced by Advanced BioNutritionals^(R)^, in a 65-year-old Caucasian female who presented with accelerated hypertension, hypokalemia, metabolic alkalosis and adrenergic symptoms. Cessation of the licorice-containing supplement resulted in complete resolution of the patient's hypertension, symptoms and abnormal lab values. To our knowledge this is the first reported case of AME from this supplement, and the first to describe accompanying hyperadrenergic symptoms.

**Conclusions:**

Glycyrrhizic acid is increasingly being found in unregulated nutritional supplements and has the potential to induce a reversable syndrome of AME. Acquired AME should be suspected in individuals who present with hypertension along with hypokalemia, metabolic alkalosis and low plasma renin and serum aldosterone levels.

## Background

Cases of hypertension with hypokalemia and metabolic alkalosis are typical for syndromes of excessive mineralocorticoid receptor stimulation, the most classic of which is primary aldosteronism. The syndrome of apparent mineralocorticoid excess (AME), or pseudo-hyperaldosteronism, is characterized by hypertension with hypokalemia with low plasma renin and serum aldosterone levels. This rare inherited syndrome has been reported in individuals found to have mutations in 11β-hydroxysteroid dehydrogenase type 2 (11βDSH-2). 11βDSH-2 is expressed in many tissues which express mineralocorticoid receptors (MR), including the distal nephron, salivary glands, skin, and select regions of the brain such as the paraventricular nucleus [1–4]. Cortisol has equal binding affinity to MR to that of aldosterone [5], but within the distal nephron it is rapidly converted by 11βDSH-2 to the inactive metabolite cortisone, preventing cortisol mediated MR activation. Loss-of-function of 11βDSH-2 increases local cortisol levels, increasing MR activation. In the distal nephron, MR activation increases expression of the epithelial sodium channel (ENaC), leading to sodium and water retention.

Licorice consumption is a well described secondary cause of hypertension by inducing acquired AME [6, 7]. Licorice root contains high concentrations of glycyrrhizic acid, which inhibits 11βDSH-2 causing acquired AME syndrome. Glycryrrhizic acid is also an antagonist of the other 11β hydroxysteroid dehydrogenase, 11βDSH-1, expressed within the liver, pancreas, adipose tissue and throughout the brain where it acts as a bidirectional catalyst for the interconversion of cortisone and cortisol. Glycyrrhizic acid is found in a variety of candies, chewing tobacco, traditional medicines, and increasingly in nutritional supplements. We report a case of acquired AME due to ingestion glycyrrhizic acid containing Advanced Bionutritionals^(R)^ Advanced Liver Support nutritional supplement. Our patient’s presentation is unique as they also displayed hyperadrenergic symptoms, which have not been reported to our knowledge in other AME cases.

## Case report

A 65-year-old Caucasian female with a past medical history of asthma presented to the emergency department after noting a systolic blood pressure of 220 mmHg at home. Earlier in the day she had been diagnosed with hypertension by her primary care physician and prescribed Olmesartan 20 mg daily. In the emergency room she complained of a mild headache and chest tightness. She also noted symptoms of anxiety, tremor, diaphoresis, and agitation which had been present for the past few months. Her medications included olmesartan 20 mg daily, budesonide-formoterol inhaler and fluticasone nasal spray needed, and an over-the-counter nutritional supplement.

She had a blood pressure of 197/89 mmHg, a heart rate of 104 beats/min, and a respiratory rate of 13 breaths/min. She appeared in moderate distress. She had a benign cardiac and respiratory exam and had no focal neurologic deficits. An EKG showed normal sinus rhythm without evidence of ischemia. Serial high-sensitivity troponins were completed which were in normal reference range. Her complete blood count and serial high sensitivity troponins were in the normal reference range. Her serum sodium was 141 mmol/L, potassium 3.1 mmol/L, chloride 101 mmol/L, bicarbonate 30 mmol/L, blood urea nitrogen of 13 mmol/L, and creatinine of 0.7 mg/dL with an estimated glomerular filtration rate of 96 mL/min/1.73 m^2^. Bloodwork completed 4 months prior showed sodium 133 mmol/L, a potassium level of 4.6 mmol/L and bicarbonate level of 27 mmol/L. The patient was diagnosed with hypertensive urgency and 5 mg of amlodipine daily was initiated.

Two days later the patient attended outpatient nephrology follow up and was found to be persistently hypertensive with a blood pressure of 188/86 mmHg despite a regimen of olmesartan 20 mg daily and amlodipine 5 mg daily. She again noted mild headache and persistent adrenergic symptoms of tremors, anxiety and agitation. She was diagnosed with accelerated hypertension. On investigation for secondary causes of hypertension found she was euthyroid with a TSH of 2.24 µIU/mL (reference range 0.4–4.2 µIU/mL) and free T4 of 1.18 ng/dL. Her serum fasting free metanephrines and normetanephrines were normal at 22.9 pg/mL (reference range 0–88 pg/mL) and 51.5 pg/mL (reference range 0–285.2 pg/mL). Her plasma renin activity was reduced at 0.437 ng/mL/hr (reference range 0.167–5.38 ng/mL/hr) and her serum aldosterone level was less than 1 ng/dL (reference range 4–10 ng/dL). olmesartan had been discontinued prior to drawing renin and aldosteorne levels.

The patient's presentation of hypertension with hypokalemia and metabolic alkalosis, and low plasma renin and serum aldosterone levels suggested the diagnosis of AME or Liddle syndrome. Liddle syndrome was excluded given her prior normal baseline metabolic panels. The patient was noted to be taking 4 tablets daily of Advanced Liver Support produced by Advanced Bionutritionals^(R)^ (Nacros, Georgia) for the prior 4 months, which contains amongst its ingredients 250 mg of Licorice root extract per tablet for a total daily dose of 1,000 mg. The patient was diagnosed with hypertension secondary to glycryrrhizic acid induced acquired AME. Her supplement was stopped, and she had resolution of her hypertension, adrenergic symptoms, hypokalemia and her metabolic alkalosis on follow-up 2 weeks later, and her blood pressure medications were discontinued without further hypertension. She remained without hypertension of metabolic abnormalities on 3 month and 1 year follow-up. Confirmatory testing for AME via a 24-h urinary cortisol/cortisone ratio was not completed given the patients return to normotension along with her resolution of hypokalemia and metabolic alkalosis.

## Discussion

We present a case of acquired AME secondary to ingestion of Advanced BioNutritionals^(R)^ Advanced Liver Support supplement. To our knowledge, this is the first reported case of acquired AME from this licorice root containing nutritional supplement. Our patient was consuming 1,000 mg daily of licorice root extract containing at least 20% glycryrrhizic acid, the pharmacologically active component of licorice root. Cases of licorice induced AME are rare in the United States, as the FDA regulates the maximum glycyrrhizin content which can be added to food products. Furthermore, many nutritional supplements which contain licorice are deglycyrrhizinated, free of the active ingredient which causes AME. However as in this case, there are still a variety of glycyrrhizin containing nutritional supplements available on the market (Table 1). Acquired AME has been observed with doses of glycryrrhizic acid greater than 217 mg/day [6], although there is significant variability among individuals in their sensitivity to licorice mediated AME. One proposed explanation for differences in susceptibility to glycyrrhizic acid is genetic variations in the epithelial sodium channel (ENaC) [7]. ENaC is responsible for MR-regulated sodium retention within the distal nephron, and individuals who have more prolific ENaC activity or expression may be more suspectable to glycyrrhizic acid induced hypertension. As the patient's hypertension and metabolic abnormalities had rapidly resolved, confirmatory testing for AME via 24 h urinary cortisol/cortisone ratio testing was not performed.

**Table 1:** Examples of nutritional supplements which contain licorice root on the market

Supplement	Total licorice root per serving	Marketed Indication
Advanced liver support by Bionutrionals^(R)^ (Nacross, GA)	1000 mg (4 capsules per serving)	Liver support
Nature's Way ^(R)^ Licorice Root (Green Bay, WI)	900 mg (2 capsules per serving)	IBS and digestive aid
Nature’s Answer ^(R)^ Licorice Root Extract (Hauppauge, NY)	2000 mg (2 mL per serving)	IBS and digestive aid
Oregan’s Wild Harvest ^(R)^ Licorice (Redmond, OR)	1350 mg (3 capsules per serving)	Endurance and vitality
PipingRock ^(R)^ Menopause Ease (Ronkonkoma, NY)	120 mg (3 capsules per serving)	Control of menopause symptoms
Gundry MD ^(R)^ Total Restore (Beverly Hills, CA)	54 mg (3 capsules per serving)	Healthy gut lining

Glycryrrhizic acid induces AME through inhibition of 11βDSH-2, a mechanism which parallels the effects of loss-of-function mutations seen in inherited AME. Glycryrrhizic acid has additional effects on cortisol metabolism, as it is also an antagonist of the other hydroxysteroid dehydrogenase isoenzyme, 11βDSH-1. 11βDSH-1 is a NADPH/NADP + mediated bidirectional catalyst of cortisol and cortisone conversion [8]. The direction of 11βDSH activity is regulated through the ratio of NADPH/NADP + , with an increase in NADPH increasing reduction of cortisone to cortisol. Its kinetics from tissue samples typically favor reduction of cortisone to cortisol, resulting in a net increase in local cortisol levels. 1βDSH-1 is expressed in the liver, adipose tissue, muscle, pancreas, and throughout the brain [2, 9] and is responsible for approximately 30% of daily cortisol production through the salvage of cortisone within the splanchnic circulation [10].

We believe our patient had hyperadrenergic symptoms due to glycryrrhizic acid mediated changes in cortisol expression and signaling within the CNS. To our knowledge, this is the first case reported of licorice induced AME to also note hyperadrenergic symptoms. Our patient complained of significant agitation, tremors, and anxiety which resolved along with her AME after cessation of her nutritional supplement. We suspect her symptoms could be secondary changes in local cortisol levels within the CNS, altering local glucocorticoid receptor and MR signaling. 11βDSH-2 is expressed in the paraventricular nucleus (PVN) of rats, and inhibition of 11βDSH-2 with glycryrrhizic acid has been shown to increase sympathetic outflow from the PVN through glucocorticoid dependent activation of MR [4]. While it remains unclear whether humans express 11βDSH-2 within our PVN, this mechanism would explain our patient’s increased adrenergic symptoms. A second explanation for our patient’s adrenergic symptoms could be through inhibition of 11βDSH-1. As 11βDSH-1 is a bidirectional catalyst, its inhibition could also result in increased local cortisol levels based upon the local metabolome and balance of cofactors NADPH/NADP + . Further investigation of the endocrine and paracrine effects of 11βDSH-1 and 11βDSH-2 within the CNS is required and could lead to a better understanding of the body's regulation and role of steroids in the brain.

## Conclusions

Glycyrrhizic acid ingestion can induce an acquired AME syndrome, which in this case presented in tandem with previously undescribed hyperadrenergic symptoms. AME and other excess mineralocorticoid activity syndromes should be suspected when hypertension is present with metabolic alkalosis and hypokalemia.

## Data Availability

Not applicable.

## Abbreviations

AME: Apparent mineralocorticoid excess
11βDSH-1: 11β Hydroxysteroid dehydrogenase type 1
11βDSH-2: 11β Hydroxysteroid dehydrogenase type 2
ENaC: Epithelial sodium channel
HPA: Hypothalamic pituitary adrenal gland axis
MR: Mineralocorticoid receptor
